# An Unusual Presentation of Neurosarcoidosis in a 64-Year-Old Man: A Case Report

**DOI:** 10.7759/cureus.60146

**Published:** 2024-05-12

**Authors:** Kalashree Gopal, Jeremiah Howard, Shyamalee Ramaraj, Ahaj H Shroff, Christopher Gamard

**Affiliations:** 1 Internal Medicine, Alabama College of Osteopathic Medicine, Dothan, USA; 2 Neurology, Alabama College of Osteopathic Medicine, Dothan, USA; 3 Internal Medicine, Mobile Infirmary Medical Center, Mobile, USA

**Keywords:** diplopia, hilar lymphadenopathy, uveitis, rare cause of dysphagia, antidiuretic hormone and neurosarcoidosis, atypical sarcoid

## Abstract

Sarcoidosis is a multisystem granulomatous disorder with an unknown etiology that typically involves the lungs, skin, and lymph nodes, with neurological involvement being relatively rare. We discuss a case of neurosarcoidosis in a 64-year-old man who initially presented with unexplained cognitive impairment, insomnia, hyponatremia, paresthesias, and weight loss and later developed uveitis, diplopia, and dysphagia. Ultimately, findings of hilar and mediastinal lymphadenopathy on chest computed tomography (CT) resulted in bronchoscopy, which led to the diagnosis. This case highlights a rare presentation of sarcoidosis with an unusual constellation of symptoms. We discuss the difficulty involved in diagnosing this disorder as well as its highly variable course.

## Introduction

Clinical manifestations of sarcoidosis can vary widely. It can be relatively asymptomatic or present with symptoms such as dry cough, weight loss, fatigue, night sweats, and erythema nodosum. Due to its nonspecific symptoms, diagnosis can be difficult, and a definitive diagnosis can only be made via pathology [1]. Sarcoidosis has been linked to various human leukocyte antigens (HLAs) and immune-related genes and is often precipitated in genetically susceptible individuals by an environmental trigger. This results in the activation of the major histocompatibility complex II (MHC II) pathway, with antigen-presenting cells (APCs) activating T cells. Following antigen presentation, activated T cells release cytokines and chemokines, resulting in granuloma formation. In sarcoidosis, the granuloma is typically noncaseating [2]. Lymphadenopathy is often present, and while peripheral lymphadenopathy may be present in a minority of cases, the hilar and mediastinal lymph nodes are most commonly affected [3].

The lungs are involved in over 90% of sarcoidosis cases, which manifest as dry cough, dyspnea, and fatigue and can chronically lead to lung fibrosis and respiratory failure. Ocular involvement occurs in over 40% of patients, which can present as pain, photophobia, and hyperemia. Neurological involvement occurs in less than 10% of sarcoid patients, making it quite an uncommon presentation [1]. Common clinical manifestations of neurosarcoidosis include cranial neuropathy (with facial nerve palsy being the most common followed by optic neuropathy), leptomeningeal inflammation, cognitive dysfunction, and peripheral neuropathy [4]. We present a case of neurosarcoidosis with unusual features and additional pulmonary and ocular manifestations. 

## Case presentation

A 64-year-old man presented to his primary care physician two weeks after a three-day hospitalization for persistent vomiting and dehydration. During his hospitalization, he was found to be significantly hyponatremic, and at the time, this was concluded to be due to hypovolemia from vomiting with primarily free water replacement. He received intravenous (IV) fluids with moderate improvement in his sodium levels and was subsequently discharged. The patient additionally reported a one-month history of unexplained weight loss, insomnia, and malaise to his primary physician, as well as a two-week history of balance difficulty, cognitive impairment, and lower abdominal paresthesias.

Over the following weeks, the patient remained significantly hyponatremic. This persisted for approximately six weeks, and significant improvement was only seen once he was placed on a restrictive fluid regimen by his primary physician. Over the next two months, the patient also experienced continued weight loss and significant worsening of other symptoms, including cognitive impairment, insomnia, and weakness. His paresthesias additionally spread to his upper and lower extremities.

As his symptoms worsened, the patient underwent an extensive workup with his primary care physician as well as multiple specialists. Prior chest X-ray, head CT, spinal magnetic resonance imaging (MRI), and brain MRI had all been unremarkable. An RPR/VRDL (rapid plasma reagin/venereal disease research laboratory) screening had been performed due to his cognitive impairment and neurologic changes which ruled out neurosyphilis. Due to his weight loss and weakness, a cosyntropin stimulation test was performed, which did not suggest adrenal insufficiency. An extensive serologic evaluation for thyroid dysfunction, vitamin deficiencies, hyperammonemia, or any other metabolic derangement was also largely unremarkable. An esophagogastroduodenoscopy (EGD) was performed to rule out malabsorption syndrome as the cause of his weight loss. The EGD only showed mild nonerosive antritis, likely secondary to daily aspirin use. Of note, he was found to have uveitis at an eye exam approximately six weeks after his initial presentation. Following this finding, differential considerations included sarcoidosis, lymphoma, tuberculosis, Lyme disease, ankylosing spondylitis, etc.

**Figure 1:**
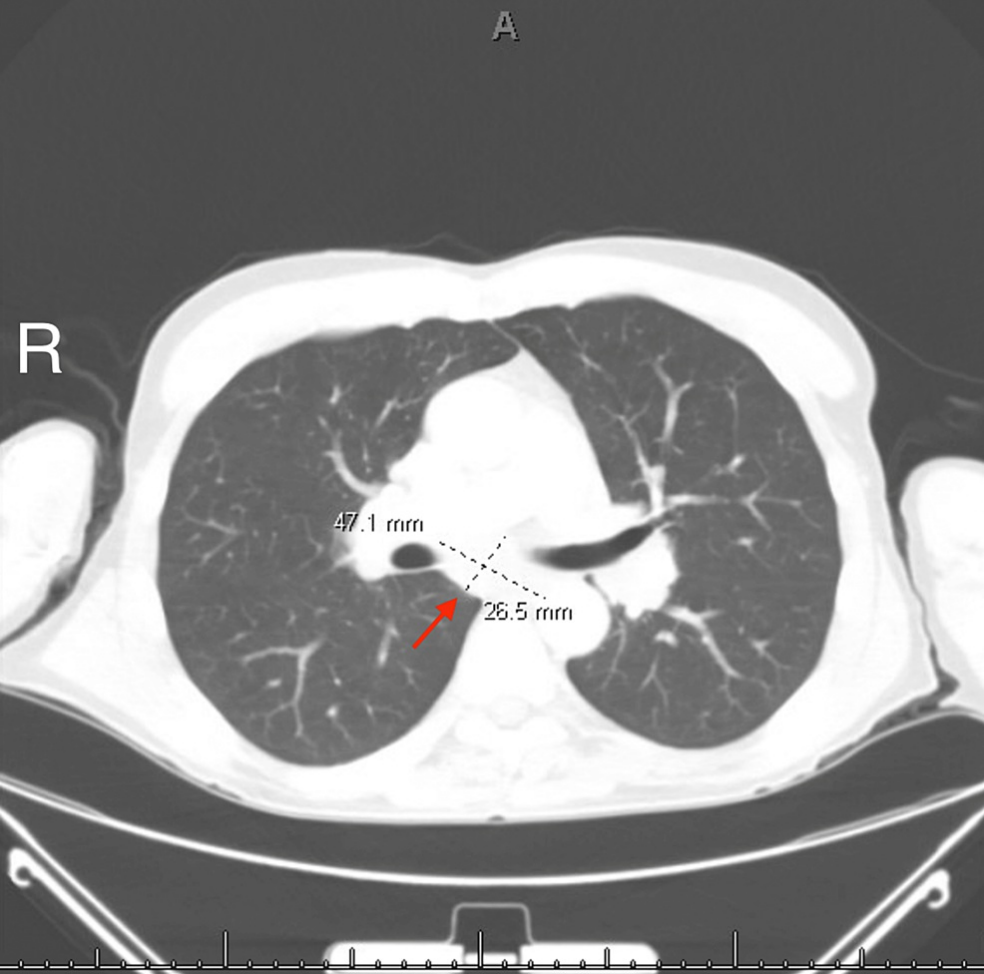
CT of the chest depicting an enlarged subcarinal lymph node measuring 4.7 cm x 2.7 cm. CT: computed tomography.

**Figure 2:**
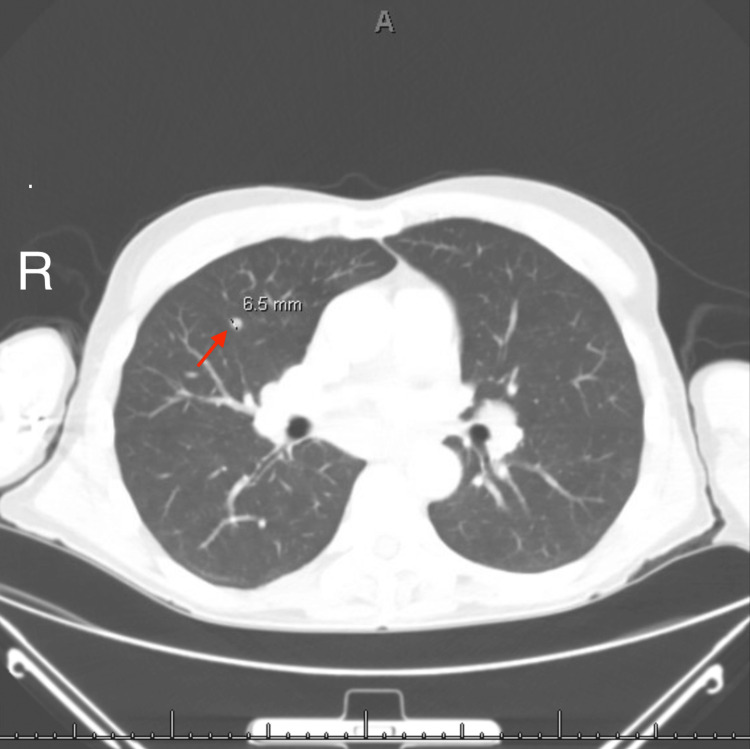
Chest CT depicting an intrapulmonary lymph node measuring 6.5 mm. CT: computed tomography.

Approximately two months after symptom onset, our patient presented to the emergency department with a three-day history of dysphagia. Since he had eaten nothing solid for the previous three days with little liquid intake, his family was concerned about dehydration. In the hospital, a computed tomography (CT) scan of the chest with contrast was performed, which showed hilar and mediastinal lymphadenopathy concerning lymphoma (Figures 1, 2). CT scans of the neck and abdomen were both negative for lymphadenopathy. A bronchoscopy was also performed during the patient’s hospital stay, which was negative for malignancy but demonstrated noncaseating granulomatous disease consistent with sarcoidosis. A lumbar puncture was also done, which showed a cerebrospinal fluid (CSF) protein of 134 mg/dL, a leukocyte count of 30 per mm^3^, and no other abnormalities. During his hospitalization, he also developed diplopia, light sensitivity, eye pressure, and weakness of his lips, making it difficult for him to suction and consume liquids. Due to the granulomatous changes seen on bronchoscopy and his uveitis, the patient was started on prednisone 40 mg per day for presumed sarcoidosis. His dysphagia improved almost immediately after starting steroids, and his appetite and fluid intake improved swiftly as well.

The patient was seen by outpatient neurology approximately one week after discharge. Since his presentation, CSF findings, and significant improvement with steroid treatment were all consistent with central nervous system involvement, he was placed on a more aggressive steroid course that was eventually tapered. He was later started on adalimumab, with continued improvement of his appetite, weight gain, insomnia, weakness, vision, and cognitive impairment over the following months.

## Discussion

Sarcoidosis most commonly involves the pulmonary system, skin, eyes, and lymph nodes. There are genetic as well as environmental components involved in its pathogenesis, and those lead to the activation of the MHC II pathway, ultimately resulting in T-cell activation and granuloma formation. Due to their autoimmune nature, steroids and other immunosuppressive agents are the cornerstone of treatment [2].

Neurosarcoidosis is relatively rare, with neurological manifestations only presenting in about 10% of patients [1]. More common clinical phenotypes of neurosarcoidosis include meningitis, cranial neuropathy, myelopathy, sellar disease, parenchymal disease, encephalopathy, neuropsychiatric disease, and peripheral neuropathy. The most frequently affected cranial nerves are the optic, facial, and vestibulocochlear nerves [5].

Our patient did not initially present with cough, dyspnea, or any other signs of pulmonary involvement and previously had a normal chest X-ray. He did present with cognitive impairment, insomnia, and fatigue, which are relatively common in patients with sarcoidosis and/or neurosarcoidosis [6,7]. He was also discovered to have uveitis during an eye exam, which is a relatively common symptom of sarcoidosis and presents in about 40% of sarcoid patients [1].

Our patient additionally presented with persistent hyponatremia that only improved with restrictive fluid intake. This is a rare symptom of neurosarcoidosis that has been reported in case studies, although the exact mechanism and whether it involves the syndrome of inappropriate antidiuretic hormone (SIADH) secretion are still unclear. There have additionally been reports of diabetes insipidus in sarcoid patients, and the pathology behind how sarcoidosis can lead to deficiency and excess of antidiuretic hormone (ADH) is not well understood [8]. This is a topic that would benefit from further research.

Neuro-ophthalmologic involvement in sarcoidosis is also considered quite rare, occurring in about one-third of patients with neurosarcoidosis and therefore 3-4% of overall sarcoid patients. The major symptoms of neuro-ophthalmic involvement are loss of vision and diplopia, and diplopia is typically due to involvement of the oculomotor, trochlear, and/or abducens nerves [9]. In our patient’s case, he developed double vision approximately two months into the course of his initial presentation. This symptom also seemed to persist in our patient even after extensive steroid therapy and improvement in almost all other aspects of his functioning. Prismatic lenses were provided to our patient to correct his double vision and improve functioning.

Dysphagia is another highly unusual symptom of sarcoidosis. It may be a result of cranial nerve involvement, invasion of the enteric nervous plexus with lower motor neuron involvement, direct esophageal skeletal muscle infiltration, or mechanical obstruction of the esophagus by mediastinal lymph nodes [10]. Our patient had a negative EGD study only 10 days prior to his hospital presentation with dysphagia. He did have mediastinal and hilar lymphadenopathy on chest CT, although these lymph nodes were not large enough to cause obvious compression of the esophagus. Given that he did additionally have oral motor issues with sucking, this suggests that he may have had cranial nerve involvement. Dysphagia is an important clinical presentation, despite its rarity due to the potential for malnutrition and dehydration, and is therefore worth highlighting. Fortunately, our patient did have rapid improvement in his swallowing with steroid therapy.

## Conclusions

Sarcoidosis is a complex disorder that is often difficult to diagnose as its presentation is often generalized and affects multiple organ systems. The pulmonary system is the most commonly involved, and the cutaneous and lymphatic systems are frequently affected as well. Less common presentations that do not involve these systems may make sarcoidosis especially hard to diagnose. In addition, cases with predominantly neurological features and rarer complications such as dysphagia and hyponatremia can pose a significant diagnostic challenge. This report discusses an unusual presentation of sarcoidosis in the hopes of better understanding and earlier detection of this complex disease. We hope to emphasize the importance of keeping sarcoidosis high on a differential list when there is a vast array of systemic as well as organ-specific symptoms that may not appear interconnected.
